# Prenatal Self-Evaluation Questionnaire in Peruvian Women: Analysis Through the Psychometric Network

**DOI:** 10.1089/whr.2025.0003

**Published:** 2025-06-19

**Authors:** Antonio Serpa-Barrientos, Juan Jose Gabriel Artica Martinez, Enrique Giovanni Pérez-Flores, Jacksaint Saintila

**Affiliations:** ^1^ Department of Psychology, Universidad Nacional Mayor de San Marcos, Lima, Peru.; ^2^ Department of Psychology, Universidad Privada del Norte, Lima, Peru.; ^3^ School of Medicine, Faculty of Health Sciences, Universidad Señor de Sipán, Chiclayo, Peru.

**Keywords:** pregnancy, self-assessment, psychometrics, reliability, validity, network analysis

## Abstract

**Background:**

Lederman designed the prenatal self-evaluation questionnaire, which is a widely used tool for evaluating the psychological well-being and health perception of women during pregnancy. However, its structure and reliability may vary according to the population and cultural context in which it is applied. In Peru, no exhaustive studies have been conducted to validate the psychometric properties of this questionnaire.

**Objective:**

Assess the psychometric properties of Lederman-designed prenatal self-evaluation questionnaire in a sample of Peruvian women using the network approach (BootEGA).

**Methods:**

An instrumental investigation was conducted involving 790 women whose ages ranged from 18 to 45 years (*M* = 23.87, SD = 6.76). The network approach was used to analyze the data and evaluate the structure of the questionnaire.

**Results:**

The results obtained revealed the presence of a network structure consisting of five dimensions. These dimensions showed stability levels (0.70) and optimal average loads (0.15), supporting the idea that Lederman designed the prenatal self-evaluation questionnaire be composed of five different dimensions. Furthermore, structural consistency showed that the questionnaire is accurate and stable, with estimates <0.99.

**Conclusion:**

Using the network method, the psychometric robustness of the Lederman-designed prenatal self-evaluation questionnaire was satisfactorily validated in the sample of Peruvian women. These findings reinforce the questionnaire’s relevance as a reliable tool for assessing psychological well-being during pregnancy, providing valuable insights for maternal health professionals.

## Introduction

Pregnancy, recognized as a critical period in a woman’s life, is a phase of integral transformation that involves not only biological changes but also profound psychological and social adjustments.^1^ Biologically, this stage is characterized by significant hormonal and metabolic changes that prepare the woman’s body for pregnancy and childbirth, which, in turn, can affect various bodily and emotional functions.^2^ At the intrapsychological level, pregnancy is a time of intense emotional and cognitive restructuring. Pregnant women may experience a wide range of emotions, from joy and anticipation to anxiety and fear, as they adjust to their new identity as mothers.^3^ At the interpersonal level, pregnancy also involves a readjustment in relationships. This may include changes in couple dynamics, the relationship with the extended family, and the social circle.^4^ These changes and adjustments, although natural and necessary, can result in temporary imbalances and alterations in the pregnant woman’s identity.^3^ Such imbalances may manifest as mood fluctuations, anxiety about the baby’s health and well-being, concerns about motherhood and parental competence, and changes in self-image and self-esteem.^1^ Therefore, recognizing and addressing these multidimensional aspects can help ensure that pregnancy is a positive and enriching experience for the expectant mother.

In the Peruvian context, there is a challenge to address prenatal care from a broader approach that includes the physical and emotional experiences of pregnant women.^5^ This arises because health professionals tend to focus more on the well-being of the fetus, often neglecting the physical and psychological changes that women experience during pregnancy.^6^ Despite the importance of fetal well-being, it is essential that health care providers also consider the complex psychosocial changes that mothers go through. The lack of screening tools specific to the Peruvian context limits the ability of health professionals to conduct comprehensive assessments and provide adaptive support. One proposed solution is the development of an accessible and efficient screening tool that would allow nurses and other health professionals to provide personalized interventions to help women adjust to the psychological challenges of pregnancy. This comprehensive approach would not only improve the quality of prenatal care but also promote a healthier and more satisfying pregnancy experience for women in Peru.

Several researchers have created questionnaires with the aim of studying aspects of psychosocial adaptation of pregnant women in different cultural contexts,^7–9^ including the Lederman Prenatal Self-Evaluation Questionnaire (PSEQ).^10^ This questionnaire is composed of seven key psychosocial dimensions that influence adjustment to pregnancy, as described by Lederman and Weis.^11^ These dimensions are: (a) acceptance of pregnancy, (b) identification with the role of the mother, (c) the quality of the relationship with the mother, (d) the quality of the relationship with the partner, (e) preparation for childbirth and management of fear of pain and loss of control during labor, and (f) concern for personal well-being and that of the baby.

This instrument was adapted and validated in different contexts. For example, in China, the instrument was tested in a convenience sample of 600 pregnant women, where a short version of 35 items was developed, showing good psychometric properties.^1^ Similarly, the validity and reliability of the PSEQ were analyzed in 399 pregnant women in the northeastern region of Brazil, showing adequate psychometric properties, indicating that it is a valid and reliable tool for assessing psychosocial adaptation to pregnancy in pregnant women in the Brazilian context.^12^ Likewise, the instrument shows good validity and reliability in countries such as Turkey,^13^ Spain,^14^ and Japan.^15^ In addition, this tool is used in clinical research to study how mothers adapt psychosocially in different settings, including prenatal care in high- and low-risk hospitalized mothers,^16^ in prenatal education,^17^ and in anxiety situations,^18^ among other contexts.

In Peru, the validity and reliability of the questionnaire have not yet been established, since it has not been culturally adapted to fit the specificities of the Peruvian population. This lack of cultural adaptation represents a significant barrier to its application in clinical practice and research in the Peruvian context. Without a version of the questionnaire that adequately reflects the cultural and social particularities of the country, health professionals and researchers face limitations in their ability to assess maternal psychosocial adaptation accurately and effectively; this could lead to an incomplete or biased understanding of the needs and experiences of Peruvian mothers during pregnancy. Therefore, it is important to carry out a process of cultural adaptation of the questionnaire to ensure that the data collected are relevant and applicable to the Peruvian population, thus improving the quality of prenatal care and maternal research in the country. Consequently, the aim of this research was to examine the psychometric properties of the PSEQ, considering the psychometric network as an analysis approach, specifically the exploratory graphical analysis (EGA).

## Materials and Methods

### Participants

A cross-sectional observational and instrumental design study was carried out. All participants were selected by nonprobability purposive sampling. The data were collected in the Lima area and in the Constitutional Province of Callao during the months of September and December 2023. Women whose mothers were already deceased were excluded because the PSEQ involves the relationship with the woman’s mother. A total of 790 pregnant women were evaluated, and it stands out that most of the women surveyed are experiencing their first pregnancy (62.4%), belong to nuclear families (56.1%), and have a relationship with their mothers (92.9%). In addition, there is a predominance of cohabitants (60.4%) and a varied educational level, from incomplete primary school to complete university studies. The occupation reveals that the majority are neither studying nor working (62.9%), while almost half have interrupted their studies or work due to pregnancy (47.5%). Significantly, approximately one-third of pregnancies are planned (33.7%). In terms of emotional support, the majority report the support of their parents (88.0%) and partners (89.9%). In addition, the report addresses sensitive issues such as psychological abuse (11.0%), physical abuse (2.3%), and sexual abuse (0.5%). Taken together, these data provide a comprehensive view of the sociodemographic dynamics of the population studied, crucial for understanding the context in which the analysis takes place (see Table 1).

**Table 1.** Descriptive Analysis of the Sociodemographic Variable

Variable	Category	*f*	%
Number of gestations	1	493	62.4
	2	184	23.3
	3	76	9.6
	4	19	2.4
	5	10	1.3
	6	6	0.8
	7	2	0.3
Family composition	Nuclear family	443	56.1
	Extended family	159	20.1
	Enlarged family	95	12.0
	Single-parent family	72	9.1
	Reconstituted family	4	0.5
	Family equivalent	17	2.2
Relationship with the mother	Yes	734	92.9
	No	56	7.1
Marital status	Single	213	27.0
	Married	99	12.5
	Cohabitant	477	60.4
	Widow	1	0.1
Lugar de residencia	South zone	103	13.0
	North zone	115	14.6
	East zone	348	44.1
	Central zone	215	27.2
	Province of Callao	3	0.4
Level of education	Incomplete elementary school	7	0.9
	Complete elementary school	10	1.3
	Incomplete high school	136	17.2
	Complete high school	287	36.3
	Incomplete technical superior	94	11.9
	Complete technical superior	87	11.0
	Incomplete university	115	14.6
	Complete university	54	6.8
Occupation	Studying	97	12.3
	Working	176	22.3
	Studying or working	20	2.5
	Not studying or working	497	62.9
Interruption of study or work due to pregnancy	Yes	375	47.5
	No	415	52.5
Planned pregnancy	Yes	266	33.7
	No	524	66.3
Emotional support from the parents	Yes	695	88.0
	No	95	12.0
Assumed parenthood	Yes	750	94.9
	No	40	5.1
Emotional support from the partner	Yes	710	89.9
	No	80	10.1
Psychological abuse	No	703	89.0
	Yes	87	11.0
Physical abuse	No	772	97.7
	Yes	18	2.3
Sexual abuse	No	786	99.5
	Yes	4	0.5

*f*, Frequency.

### Instrument

The prenatal self-evaluation questionnaire was designed by Lederman through interviews with a group of pregnant women.^10,11^ The original English version of the instrument was translated into Spanish by Armengol et al.^14^ for a longitudinal study on the representations in the process of motherhood. In this study, we used the brief version of 30 items elaborated by Artica^19^ in which a structure of five dimensions is proposed. This scale organization showed adequate fit indices (χ^2^ = 787.75, χ^2^/df = 1.99, Comparative Fit Index (CFI) = 0.979, Root Mean Square Error of Approximation (RMSEA) = .035 [0.032–0.039], Standardized Root Mean Square Residual (SRMR) = 0.038, Akaike Information Criterion (AIC) = 927.75); likewise, reliability coefficients were obtained for each of the five dimensions >0.92. The Spanish version of the 30-item questionnaire used in this study is included in the Supplemental Appendix.

### Ethical aspects

The Ethics Committee of the Instituto Nacional Materno Perinatal reviewed and approved the research protocol. Permission was obtained from the main author of the scale to adapt it to the Peruvian context.^11^ In accordance with the guidelines for translation and test adaptation.^20^ The survey was carried out including informed consent, which was given by each participant. In the introduction section of the questionnaire, the duration and procedure of the survey, the purpose of the research, the right to refuse or suspend participation, the absence of negative consequences for refusing or withdrawing, the lack of direct benefits for participating, and the guarantee of confidentiality and anonymity were detailed. Compliance with the ethical principles established in the Psychologists’ Code of Conduct was ensured.^21,22^

### Data analysis

The dimensionality of the Prenatal Self-Evaluation Questionnaire (PSEQ) was examined through Bootstrap Exploratory Graph Analysis (bootEGA), an innovative and robust approach in network psychometrics. Network analysis is a statistical method that represents relationships between variables as a network, where items (nodes) are connected by associations (edges). This approach helps identify clusters of related items, revealing the questionnaire’s underlying structure in a more data-driven manner. In this study, bootEGA was used to estimate the stability of dimensions and items in multivariate data.^23^

A parametric procedure was implemented to generate data from 500 initial replicate samples, using the GLASSO (Graphical Lasso) network estimation method, which applies a graphical selection operator with absolute minimum shrinkage to estimate the Gaussian graphical model. Additionally, the Walktrap community detection algorithm was employed to identify clusters of items, ensuring a reliable assessment of the questionnaire’s dimensional structure.^23^

Descriptive statistics calculations were performed for the PSEQ dimensions, including median, standard deviation, confidence interval, and quantile, providing an overview of the stability of these dimensions.^24^ Structural consistency was used as a measure to evaluate the stability of the dimensions, defined as the degree to which the items of a dimension show interrelation and homogeneity within the multidimensional structure of the questionnaire. This consistency is observed in the coherent grouping of communities in a psychological network.^25^ Thus, structural consistency is presented as an alternative to reliability coefficients (*e.g.,* α, ω) commonly used in factor analysis.^23,25^ Items with stability ≥0.75 and average network loadings significant for a small effect size (≥0.15) were considered acceptable.^24^

To carry out these analyses, Jamovi (version 2.3) was used for the descriptive statistics of sociodemographic data, and RStudio (version 4.3.0) with specific packages such as “psych,”^26^ “EGAnet,”^27^ and “qgraph”^28^ for the remaining analyses.

## Results

The psychological network model is distinguished by five main dimensions, in which the red nodes are concentrated around items related to maternal role acceptance and identification (items 1–8). The dimension linked to concern for one’s own and the baby’s well-being is represented by the color light blue, covering items 9–14. In turn, the green color illustrates the dimension that encompasses preparation, fear of pain, and loss of control during childbirth (items 15–22).

**FIG. 1.**
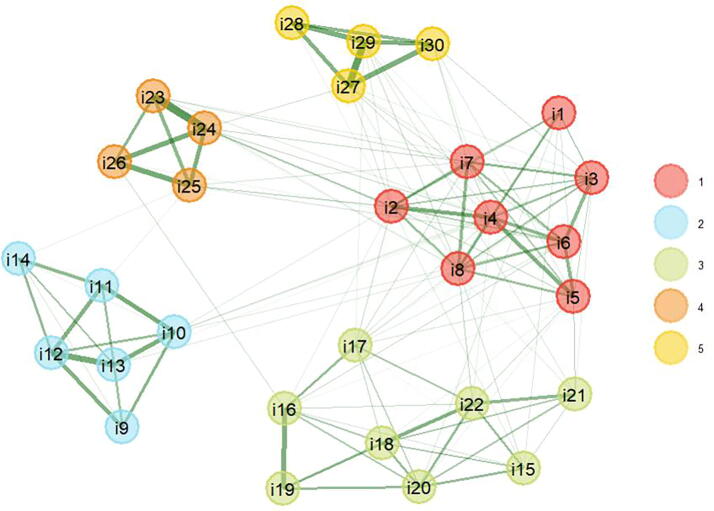
Dimensionality of the PSEQ assessed by bootstrap exploratory bootstrap graphical analysis (bootEGA). The grouping of the items in five dimensions is corroborated. PSEQ, Prenatal Self-Evaluation Questionnaire.

On the other hand, the items that assess the quality of the relationship with the mother are grouped with the orange nodes (items 23–26), while the yellow nodes are organized around the items that explore the quality of the relationship with the partner (items 27–30); for more detail, see Figure 1. In sum, the dynamic network allowed us to visualize very clearly the composition of the dimensions as reported in other studies.^19^

The five-dimensional model with 30 items exhibited a median of five dimensions, with a standard error of dimension deviation of 0. The confidence interval (95% CI [0–5]) and the lower and upper quartiles showed values identical to five. These findings indicate that the PSEQ has the same number of dimensions as the original five-dimensional theoretical matrix, as detailed in Table 2.

**Table 2.** Descriptive Statistics of PSEQ Dimensions in All Baseline Replication Samples

n.Boots	median.dim	SE.dim	CI.dim	Lower.CI	Upper.CI	Lower.Quantile	Upper.Quantile
500	5	0	0	5	5	5	5

CI, 95% confidence interval; lower, lower limit; median.dim, median; n.Boots, number of baseline replicate samples; PSEQ, Prenatal Self-Evaluation Questionnaire; SE.dim, standard deviation error; upper, upper limit; quantile, quartile.

**FIG. 2.**
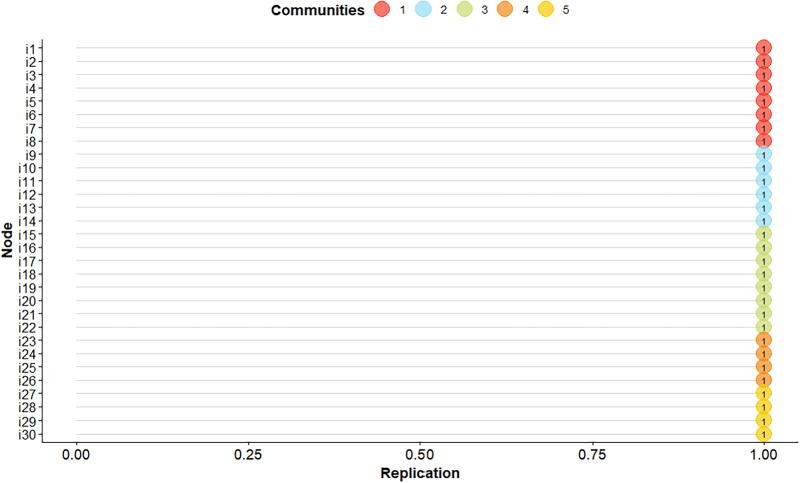
Stability of the PSEQ items.

Regarding model stability, the dimension items exhibited values that reflect complete stability (1.00), eliminating the need to explore more models to identify the most stable structure, as illustrated in Figure 2. This result confirms the consistent convergence between the items in each of the five communities.

In relation to the average item network loadings, significant parameters were observed that exceeded the small effect size (≥0.15). It should be noted that items 15, 21, and 17, belonging to the dimension of acceptance and identification with the maternal role, as well as item 1 of the dimension of concern for one’s own and the baby’s well-being, exhibited values slightly above the recommended threshold (below 0.20). However, the other items presented average loads within the optimal parameters (Table 3).

**Table 3.** Average Network Loadings of PSEQ Items Across All Baseline Replication Samples

PSEQ items	D1	D2	D3	D4	D5
i15	**0.188**	0.031	0.000	0.003	0.000
i16	**0.255**	0.049	−0.002	0.000	0.032
i18	**0.337**	0.008	0.000	0.001	0.002
i19	**0.237**	0.003	0.000	0.000	0.001
i20	**0.352**	0.015	−0.003	0.002	0.000
i21	**0.173**	0.043	0.002	0.014	0.000
i22	**0.334**	0.055	−0.005	0.003	0.000
i17	**0.166**	0.080	0.000	0.008	0.001
i1	0.025	**0.168**	0.004	0.000	0.000
i2	0.046	**0.277**	0.001	0.036	0.058
i3	0.018	**0.267**	0.022	0.042	0.021
i4	0.025	**0.382**	0.009	0.044	0.059
i5	0.096	**0.267**	0.002	0.019	0.001
i6	0.035	**0.300**	0.021	0.037	0.027
i7	0.026	**0.274**	0.014	0.041	0.045
i8	0.066	**0.301**	0.000	0.044	0.022
i9	−0.001	0.004	**0.266**	0.000	0.000
i10	0.000	0.036	**0.397**	0.002	0.005
i11	−0.005	0.001	**0.361**	0.001	0.009
i12	−0.001	0.000	**0.465**	0.000	0.000
i13	0.000	0.011	**0.401**	0.000	0.000
i14	0.000	0.003	**0.214**	0.002	0.008
i27	0.003	0.064	0.001	**0.447**	0.023
i28	0.002	0.006	0.000	**0.334**	0.000
i29	0.018	0.046	0.000	**0.457**	0.001
i30	0.002	0.057	0.004	**0.347**	0.001
i23	0.000	0.039	0.001	0.003	**0.405**
i24	0.002	0.067	0.002	0.022	**0.477**
i25	0.000	0.043	0.016	0.000	**0.366**
i26	0.027	0.005	0.001	0.000	**0.377**

Bold values indicate the primary community membership of each item based on average network loadings. Each item is assigned to the community in which it shows the highest average loading across replication samples.

## Discussion

Pregnancy, as a transcendental event in a woman’s life, involves not only physical changes but also profound psychosocial transformations that vary according to the cultural context.^2,4,5^ In Peru, where prenatal care often focuses primarily on the physical well-being of the fetus, understanding the maternal experience from a psychosocial perspective is less explored.^5^ This study seeks to fill this gap by proposing a culturally adapted tool to assess the psychosocial adaptation of Peruvian women during pregnancy. Through psychometric analysis, we aim to validate a questionnaire that accurately reflects the unique realities and experiences of pregnant women in Peru, providing a solid basis for improving prenatal care and psychosocial support in this context. Therefore, a psychometric network analysis approach was adopted, specifically EGA, a novel and robust methodology. Based on bootstrap exploratory analysis (bootEGA), which revealed a consistent and robust multidimensional structure within the PSEQ. This strongly demonstrated the validity of the instrument for assessing psychosocial adjustment during pregnancy.

The results obtained through the psychological network analysis of the PSEQ offer a revealing insight into the underlying structure of this instrument. The identification of five main dimensions, supported by the exploratory graphic analysis (bootEGA), confirms the validity of the initially proposed grouping of items. This dynamic approach allowed a clear visual representation of the dimensions, highlighting the concentration of red nodes in the acceptance and identification with the maternal role, light blue nodes in the concern for one’s own and the baby’s well-being, and green nodes in aspects related to preparation, fear of pain, and loss of control during childbirth.

The stability of the model proposed in the current study, evidenced through a median of five dimensions and a dimension standard deviation of zero, along with identical values in the lower and upper quartiles, provides strong support for the consistency and robustness of the identified structure.^23,24^ Furthermore, the complete stability of the items within the dimensions, evidenced by values of 1.00, confirms that the structure of the model is not only coherent but also consistent in the face of possible variations that could result from the exploration of alternative models.^25,28,29^ This finding underlines the reliability of the model in terms of its ability to maintain its structural integrity even in the context of different analytical configurations.

The analysis of the average loadings on the item network highlights the notable importance of certain specific items, including numbers 15, 21, and 17, which are part of the dimension of acceptance and identification with the maternal role, together with item 1, which corresponds to the dimension of concern for one’s own and the baby’s well-being. These items showed values slightly exceeding the recommended limit, indicating their possible central role in the representation of these particular dimensions. It is important to note that, despite these findings, the remaining items recorded average loadings that were within desirable limits, underscoring the overall robustness of the proposed network model.

The findings of this study reveal a remarkable and substantial divergence compared to the adaptation by Lederman and Weis,^11^ who identified seven key factors in the PSEQ scale. This innovative result suggests that the quality of relationships with both mother and partner may have a significant impact on the extent and relevance of certain factors within the scale, which is a novelty with respect to previous studies. This observation urges us to consider carefully how these relational elements might influence the configuration of the sample examined in our study.

The psychometric properties of the prenatal self-evaluation questionnaire (PSEQ) have been analyzed in various cultural contexts, revealing notable differences in its structure and item composition. Studies conducted in Mexico, Colombia, and Peru have predominantly used the 14-item version, rather than the original 79-item instrument, indicating an adaptation to Spanish-speaking populations. These modifications have included linguistic, conceptual, metric, and cultural adjustments, often validated through expert judgment to ensure content relevance. Furthermore, cross-cultural research has shown variability in the factorial structure of the questionnaire. While the original model proposed a seven-dimensional framework, only the Brazilian validation supported this structure, whereas studies in Spain and Colombia reported a reorganization of factors and item groupings. The most methodologically rigorous validations, such as those conducted in Spain and Colombia, concluded that a reduced-item version maintains the instrument’s psychometric robustness. Importantly, no studies to date have provided evidence of a unidimensional hierarchical structure, reinforcing the multidimensional nature of the construct. These findings highlight the necessity of considering cultural context when assessing the structural validity of the PSEQ and emphasize the importance of further research to standardize its use in diverse populations.

Furthermore, these findings underscore the intricate nature of the psychoaffective experience during pregnancy, highlighting how the interplay between biological, psychological, and social factors contributes integrally to a woman’s adjustment to her new maternal identity. These elements demonstrate the complexity and diversity of influences that can affect this essential process, emphasizing the importance of a holistic approach to the study of maternal adaptation. By highlighting these dynamic interactions, this study provides a deeper understanding of the maternal experience and underscores the need to consider a broader spectrum of factors when assessing women’s adjustment to pregnancy and motherhood. Similarly, this instrument is characterized as a specific, self-administered tool with proven validation and reliability, which is ideal for assessing the psychosocial adaptation of mothers. It focuses on important domains associated with pregnancy, the birthing process, and the experience of motherhood, making it an effective means of exploring relevant aspects of these stages. Its self-administered design facilitates its use, allowing pregnant women to provide valuable information about their own experiences in these key areas, which is vital for a comprehensive understanding of psychosocial processes during pregnancy and the transition to motherhood.

### Strengths and limitations

Although the cross-sectional design of our study limits the ability to assess the longitudinal response or predictive validity of the scale, it provides a solid and reliable basis for future research. This initial approach has allowed a detailed and accurate assessment of the current psychometric properties of the scale in the study population, thus establishing a fundamental starting point for its use in clinical and research contexts. The identification of this limitation highlights the need and opportunity for future longitudinal studies, which could explore intrapersonal changes over time and confirm the predictive validity of the scale. This step is essential to better understand the dynamics of the phenomena studied and to strengthen the practical application of the scale in different contexts and over extended periods.

## Conclusion

In conclusion, the findings obtained confirm and reinforce the five-dimensional structure of the PSEQ, providing a detailed understanding of the relationships between the items assessed. This innovative approach to psychometric assessment not only validates adaptation to pregnancy but also highlights the psychological complexity involved. By providing a detailed assessment through the PSEQ and psychometric network analysis techniques, this study illustrates the importance of psychosocial factors in pregnancy. These robust results have direct implications for both clinical practice and future research within the field of perinatal psychology.

## Data Availability

Data supporting the conclusions of this research will be made available in coordination with the corresponding author.

## Abbreviations Used

AIC: Akaike Information Criterion
APA: American Psychological Association
bootEGA: Bootstrap Exploratory Graph Analysis
CFI: Comparative Fit Index
CI: Confidence Interval
EGA: Exploratory Graph Analysis
GGM: Gaussian Graphical Model
GLASSO: Graphical Least Absolute Shrinkage and Selection Operator
ITC: International Test Commission
PSEQ: Prenatal Self-Evaluation Questionnaire
RMSEA: Root Mean Square Error of Approximation
SRMR: Standardized Root Mean Square Residual
