# Supplementary material for: Prenatal Self-Evaluation Questionnaire in Peruvian Women: Analysis Through the Psychometric Network
Source: Womens Health Rep (New Rochelle). 2025 Jun 19;6(1):644–51. doi: 10.1089/whr.2025.0003 (PMC12241840; doi:10.1089/whr.2025.0003)
Supplement: Supplementary Data [file whr.2025.0003_supp_data.pdf]

Appendix 1. Cuestionario de autoevaluación del período prenatal- versión de 30 ítems

Instrucciones:

A continuación, se presentan afirmaciones que han sido elaboradas para mujeres embarazadas con el propósito de describirse a sí mismas. Lea con atención cada afirmación y decida cuál de las opciones (mucho, con frecuencia, solo un poco, nada) describe mejor su forma de pensar y/o sentir. Luego marque con un aspa

(X) En la casilla de la opción elegida.

A= MUCHO
B= CON FRECUENCIA
C= SOLO UN POCO
D= NADA

#		A	B	C	D
1	Siento que criar a mi bebé será gratificante.				
2	Estoy contenta de estar embarazada.				
3	Siento que ya amo a mi bebé.				
4	Estoy feliz con este embarazo.				
5	Creo que puedo ser una buena madre.				
6	Siento que voy a disfrutar de mi bebé.				
7	Me gusta ver a otros padres e hijos juntos.				
8	Espero con ilusión dar a luz.				
9	Me preocupa que mi bebé pueda tener anomalías.				
10	Me preocupo por los problemas que mi bebé podría tener al nacer.				
11	Estoy nerviosa por las complicaciones que ocurran en el parto.				
12	Me preocupa que algo salga mal durante el parto.				
13	Me preocupa perder a mi bebé en el parto.				
14	Temo ser lastimada durante el parto.				
15	Puedo soportar bien el dolor durante el parto.				
16	Estoy informada de lo que me espera en el parto.				
17	Saber que el parto tiene que terminar me ayudará a mantener el control.				
18	Me siento preparada para lo que suceda en el parto.				
19	Sé algunas cosas que me pueden ayudar en el parto.				
20	Pienso que puedo soportar la incomodidad del parto.				
21	Tengo confianza de tener un parto normal.				

22	Tengo confianza en mí habilidad para mantener la calma durante el parto				
23	Mi madre muestra interés por mi bebé que está por nacer.				
24	Mi madre espera con ilusión a su nieto.				
25	En estos momentos, me siento bien cuando estoy con mi madre.				
26	Durante este embarazo, mi madre me da seguridad cuando tengo dudas de mí misma.				
27	Mi pareja y yo conversamos del bebé que va a nacer.				
28	Mi pareja me entiende (me calma) cuando me molesto.				
29	Mi pareja está interesado en conversar conmigo del embarazo.				
30	Puedo contar con el apoyo de mi pareja en el parto.				
